# Supplementary figures and images for: The real-time hand and object recognition for virtual interaction
Source: PeerJ Comput Sci. 2024 Jun 27;10:e2110. doi: 10.7717/peerj-cs.2110 (PMC11232578; doi:10.7717/peerj-cs.2110)

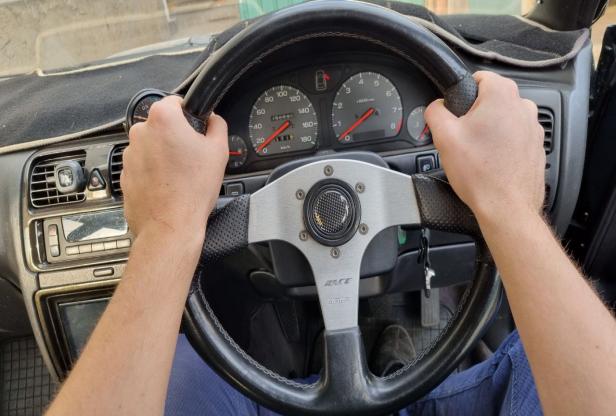

Supplement: Supplemental Information 11 [file peerj-cs-10-2110-s011.zip › keypoint_detection/The 3-Scoped Steering Wheel.v4i.yolov5pytorch/test/images/photo_2022-04-11_21-27-45_jpg.rf.710578026bc30751e0543b8a5d5807cc.jpg]

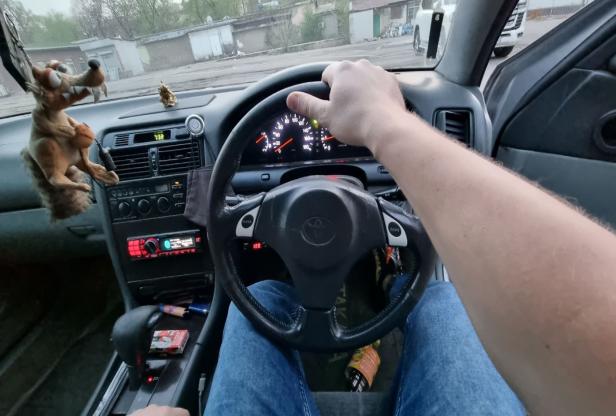

Supplement: Supplemental Information 11 [file peerj-cs-10-2110-s011.zip › keypoint_detection/The 3-Scoped Steering Wheel.v4i.yolov5pytorch/test/images/photo_2022-04-11_21-27-55_jpg.rf.3f2d2aa2cc4a4cf2f0ae6b6bf9ae2fc6.jpg]

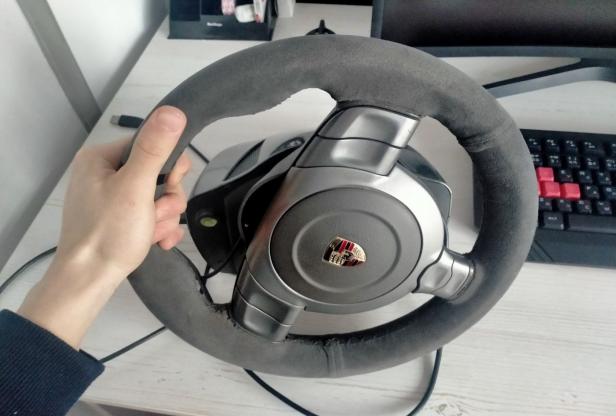

Supplement: Supplemental Information 11 [file peerj-cs-10-2110-s011.zip › keypoint_detection/The 3-Scoped Steering Wheel.v4i.yolov5pytorch/test/images/photo_2022-03-14_16-00-52_jpg.rf.29f362ce60785234972786c871ca50e6.jpg]

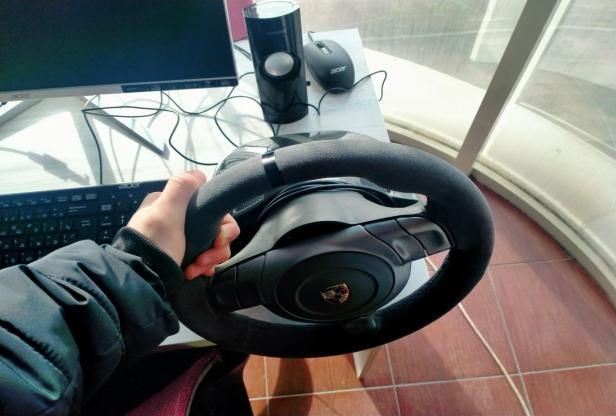

Supplement: Supplemental Information 11 [file peerj-cs-10-2110-s011.zip › keypoint_detection/The 3-Scoped Steering Wheel.v4i.yolov5pytorch/test/images/photo_2022-03-14_16-00-43_jpg.rf.c7007f0ab4389fd5ba8465597900087a.jpg]

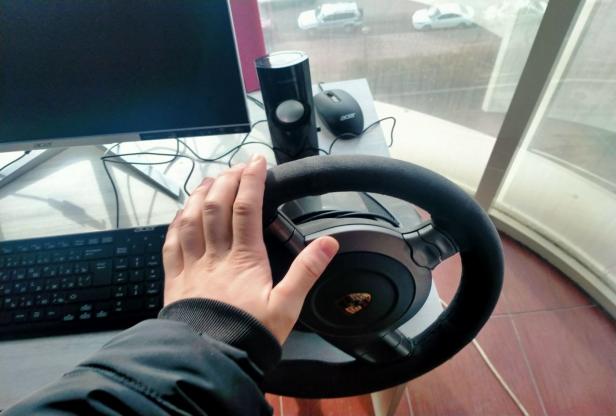

Supplement: Supplemental Information 11 [file peerj-cs-10-2110-s011.zip › keypoint_detection/The 3-Scoped Steering Wheel.v4i.yolov5pytorch/test/images/photo_2022-03-14_16-00-35_jpg.rf.68efc50cf5f9e4cce29a00c1ef4f337c.jpg]

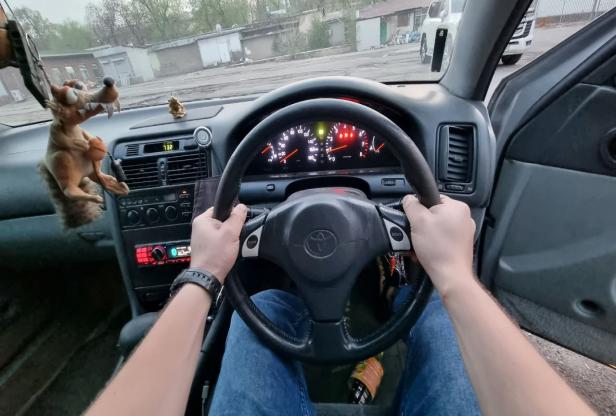

Supplement: Supplemental Information 11 [file peerj-cs-10-2110-s011.zip › keypoint_detection/The 3-Scoped Steering Wheel.v4i.yolov5pytorch/test/images/photo_2022-04-11_21-27-50_jpg.rf.c2771fcdbc4a7b3b4492dc8a3722bac9.jpg]

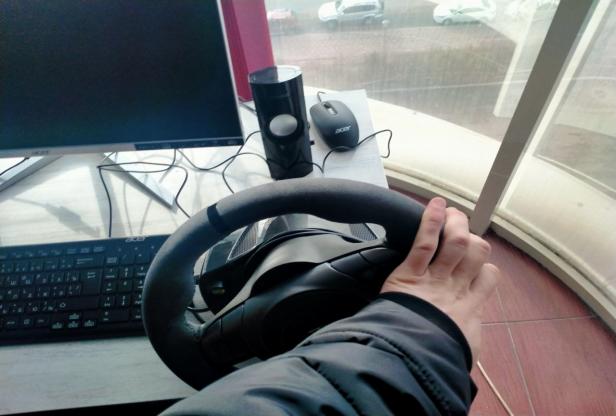

Supplement: Supplemental Information 11 [file peerj-cs-10-2110-s011.zip › keypoint_detection/The 3-Scoped Steering Wheel.v4i.yolov5pytorch/test/images/photo_2022-03-14_16-00-38_jpg.rf.e20e1faa57371acbfb4619d493576a86.jpg]

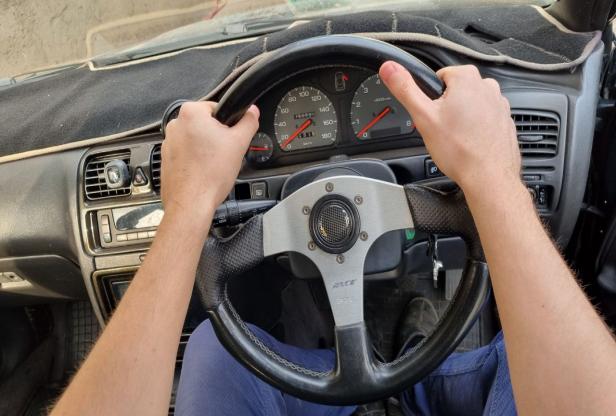

Supplement: Supplemental Information 11 [file peerj-cs-10-2110-s011.zip › keypoint_detection/The 3-Scoped Steering Wheel.v4i.yolov5pytorch/test/images/photo_2022-04-11_21-27-41_jpg.rf.75902a73882ba7263f3d12d919e988a6.jpg]

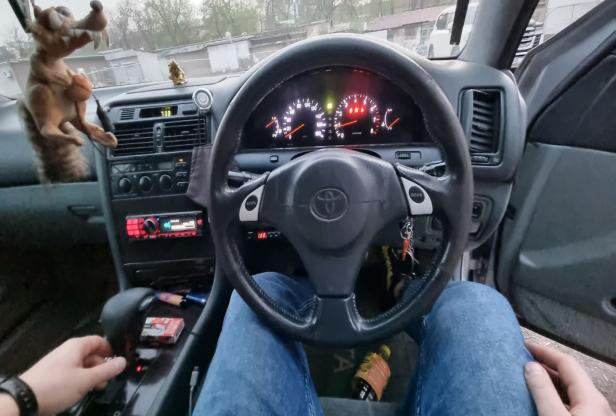

Supplement: Supplemental Information 11 [file peerj-cs-10-2110-s011.zip › keypoint_detection/The 3-Scoped Steering Wheel.v4i.yolov5pytorch/test/images/photo_2022-04-11_21-27-47_jpg.rf.c44d7d97c0e9940363a9611de2bfe848.jpg]

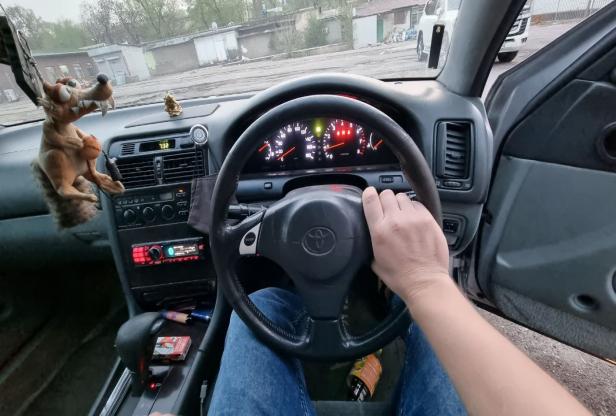

Supplement: Supplemental Information 11 [file peerj-cs-10-2110-s011.zip › keypoint_detection/The 3-Scoped Steering Wheel.v4i.yolov5pytorch/test/images/photo_2022-04-11_21-27-53_jpg.rf.0bb25f36f5919cf11b8b3f9d16e65abe.jpg]

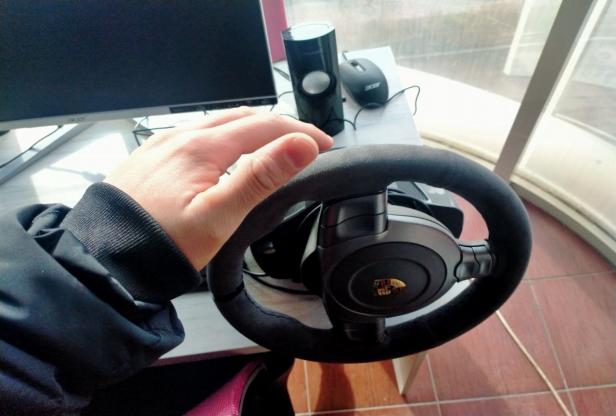

Supplement: Supplemental Information 11 [file peerj-cs-10-2110-s011.zip › keypoint_detection/The 3-Scoped Steering Wheel.v4i.yolov5pytorch/test/images/photo_2022-03-14_16-00-46_jpg.rf.0d3c57ed65f91051810b7d98d6efa94c.jpg]

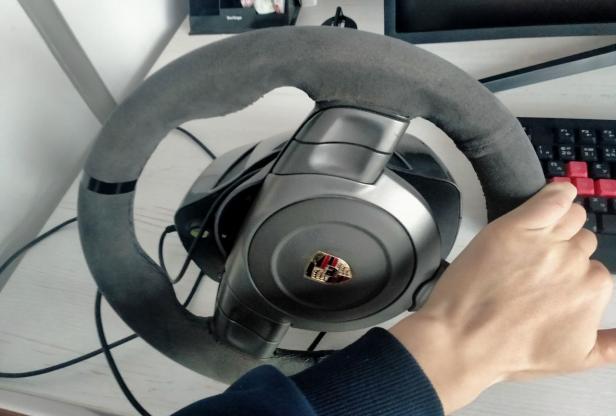

Supplement: Supplemental Information 11 [file peerj-cs-10-2110-s011.zip › keypoint_detection/The 3-Scoped Steering Wheel.v4i.yolov5pytorch/test/images/photo_2022-03-14_16-00-41_jpg.rf.eb11f9bfe11de537f46b6f25cc5ee3ce.jpg]

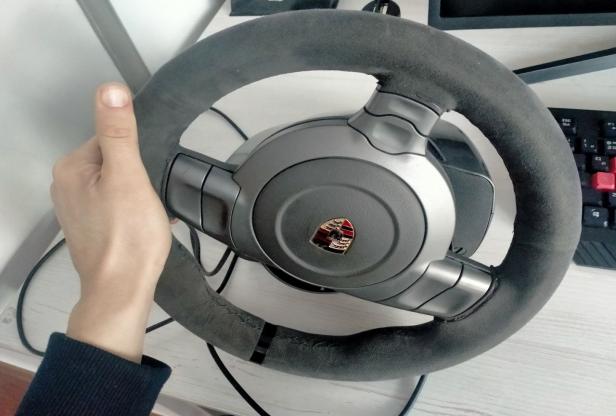

Supplement: Supplemental Information 11 [file peerj-cs-10-2110-s011.zip › keypoint_detection/The 3-Scoped Steering Wheel.v4i.yolov5pytorch/test/images/photo_2022-03-14_16-00-54_jpg.rf.b8c893512c2ce503b36e38875278471d.jpg]

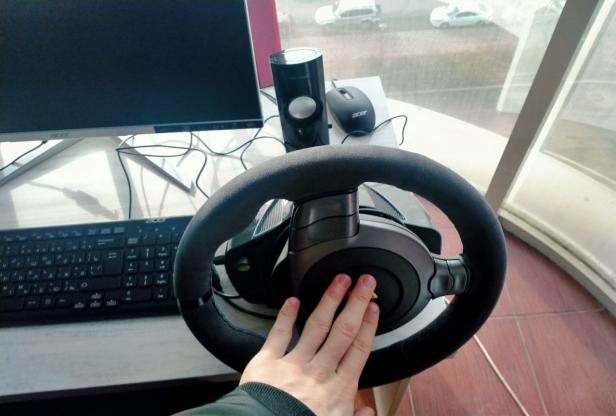

Supplement: Supplemental Information 11 [file peerj-cs-10-2110-s011.zip › keypoint_detection/The 3-Scoped Steering Wheel.v4i.yolov5pytorch/test/images/photo_2022-03-14_16-00-25_jpg.rf.13a0f43d8f7b51083984ad351157d2cf.jpg]

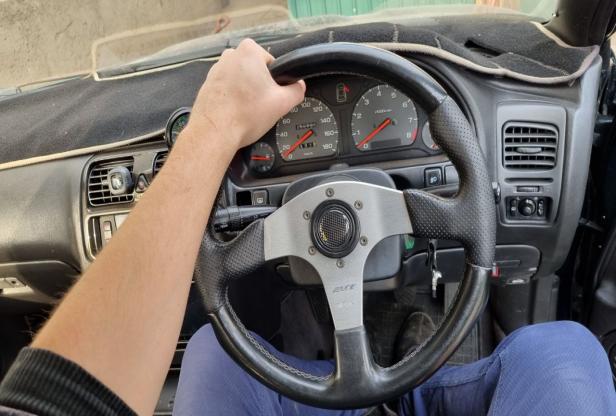

Supplement: Supplemental Information 11 [file peerj-cs-10-2110-s011.zip › keypoint_detection/The 3-Scoped Steering Wheel.v4i.yolov5pytorch/test/images/photo_2022-04-11_21-27-43_jpg.rf.2a4b60fb3784b0a502097f87dc545c68.jpg]

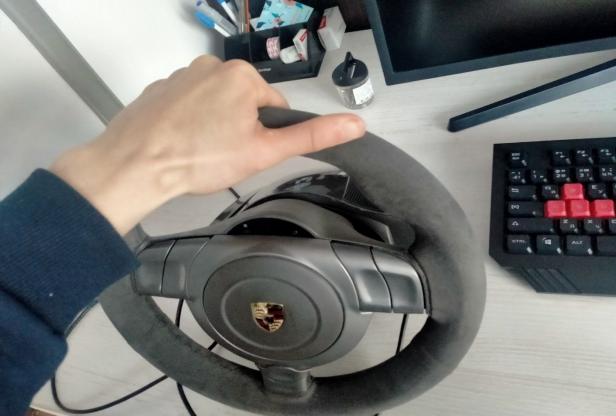

Supplement: Supplemental Information 11 [file peerj-cs-10-2110-s011.zip › keypoint_detection/The 3-Scoped Steering Wheel.v4i.yolov5pytorch/test/images/photo_2022-03-14_16-00-31_jpg.rf.4f8097e40492a33354ab9ab7a4cc0d38.jpg]

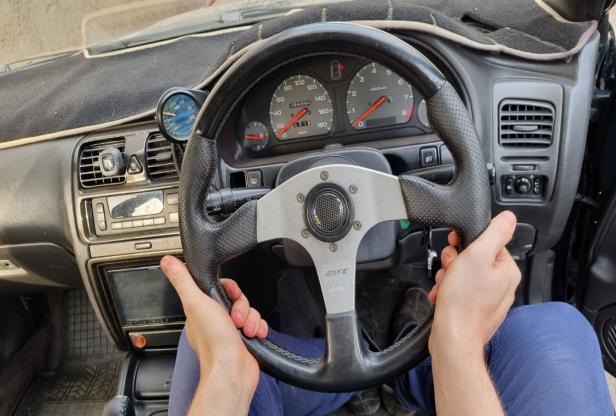

Supplement: Supplemental Information 11 [file peerj-cs-10-2110-s011.zip › keypoint_detection/The 3-Scoped Steering Wheel.v4i.yolov5pytorch/test/images/photo_2022-04-11_21-27-39_jpg.rf.690a46c6b3b41bab91b6a43be23e0bd1.jpg]

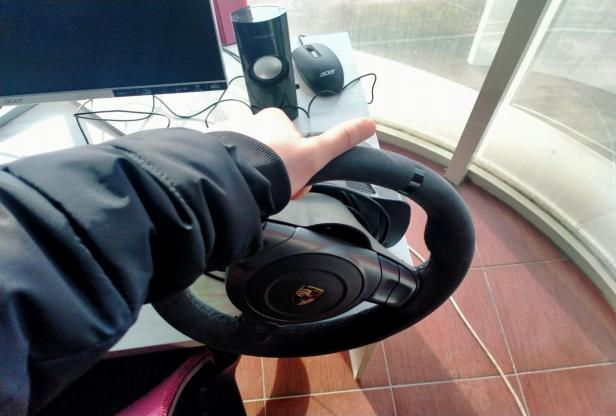

Supplement: Supplemental Information 11 [file peerj-cs-10-2110-s011.zip › keypoint_detection/The 3-Scoped Steering Wheel.v4i.yolov5pytorch/train/images/photo_2022-03-14_16-00-28_jpg.rf.36904d11520b4e47cb4d0455203b7eff.jpg]

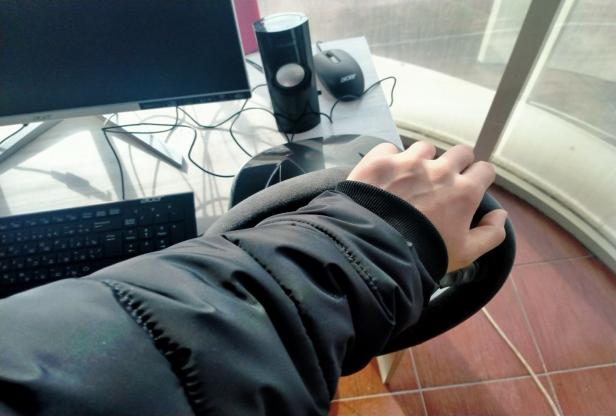

Supplement: Supplemental Information 11 [file peerj-cs-10-2110-s011.zip › keypoint_detection/The 3-Scoped Steering Wheel.v4i.yolov5pytorch/train/images/photo_2022-03-14_16-00-48_jpg.rf.6e8a916e75c1a77af28228f205df2018.jpg]

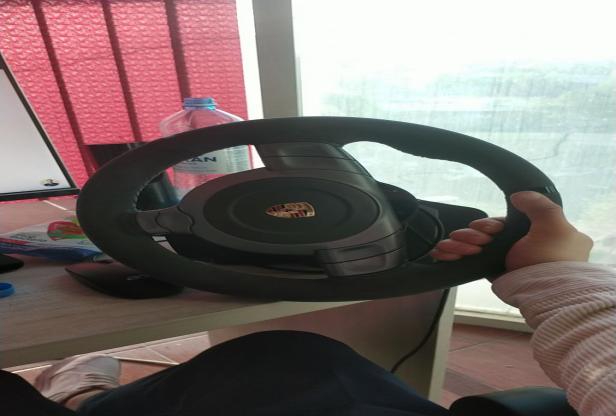

Supplement: Supplemental Information 11 [file peerj-cs-10-2110-s011.zip › keypoint_detection/The 3-Scoped Steering Wheel.v4i.yolov5pytorch/train/images/photo_2022-04-15_18-02-30_jpg.rf.75a845b413deffcc4c96e59d86a62de2.jpg]

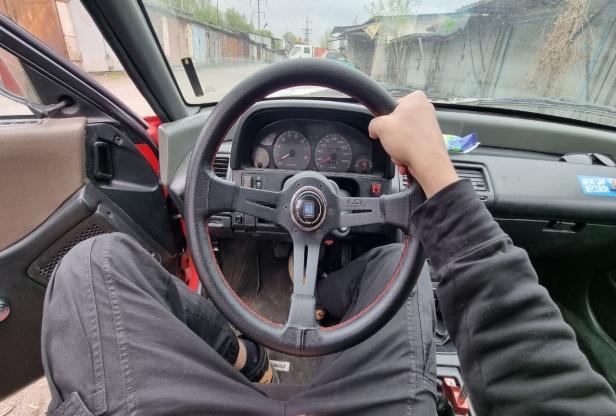

Supplement: Supplemental Information 11 [file peerj-cs-10-2110-s011.zip › keypoint_detection/The 3-Scoped Steering Wheel.v4i.yolov5pytorch/train/images/photo_2022-04-15_10-20-03_jpg.rf.53dbdc1bc17a2b07278e55c33b3d5896.jpg]

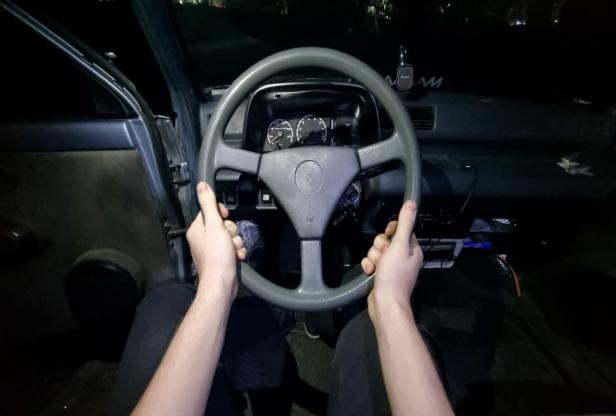

Supplement: Supplemental Information 11 [file peerj-cs-10-2110-s011.zip › keypoint_detection/The 3-Scoped Steering Wheel.v4i.yolov5pytorch/train/images/photo_2022-04-15_21-26-51_jpg.rf.0f5f28798c3f57c39f31dd0d97730df7.jpg]

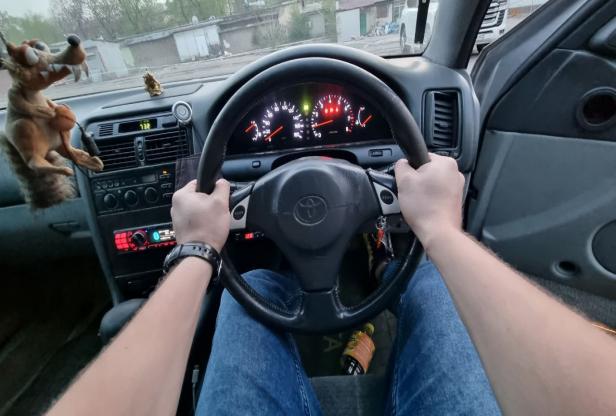

Supplement: Supplemental Information 11 [file peerj-cs-10-2110-s011.zip › keypoint_detection/The 3-Scoped Steering Wheel.v4i.yolov5pytorch/train/images/photo_2022-04-11_21-27-59_jpg.rf.4263feb5ea0d96fc307f228a841f5a07.jpg]

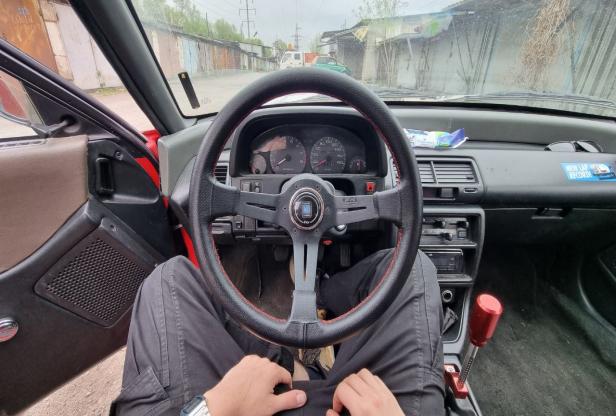

Supplement: Supplemental Information 11 [file peerj-cs-10-2110-s011.zip › keypoint_detection/The 3-Scoped Steering Wheel.v4i.yolov5pytorch/train/images/photo_2022-04-15_10-19-51_jpg.rf.1e1187dacaa5178483db186a6941c885.jpg]

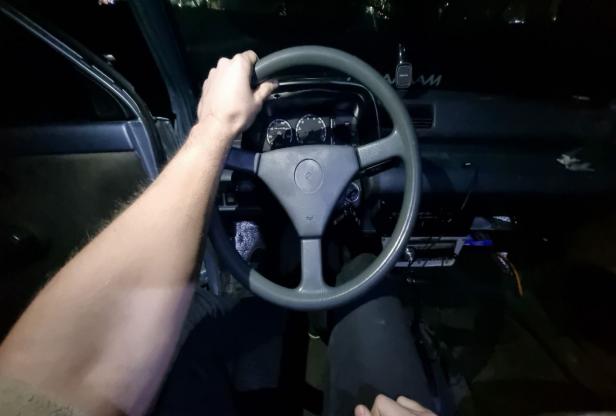

Supplement: Supplemental Information 11 [file peerj-cs-10-2110-s011.zip › keypoint_detection/The 3-Scoped Steering Wheel.v4i.yolov5pytorch/train/images/photo_2022-04-15_21-26-59_jpg.rf.45063b6923f4640e84d718eda4998f8f.jpg]

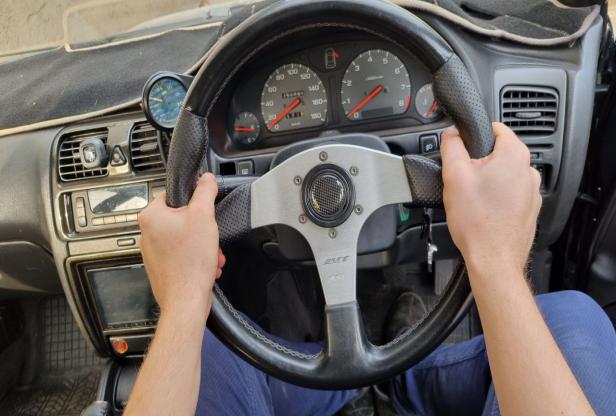

Supplement: Supplemental Information 11 [file peerj-cs-10-2110-s011.zip › keypoint_detection/The 3-Scoped Steering Wheel.v4i.yolov5pytorch/train/images/photo_2022-04-11_21-27-44_jpg.rf.9e93d6ceb8097279e7009bb46848ead2.jpg]

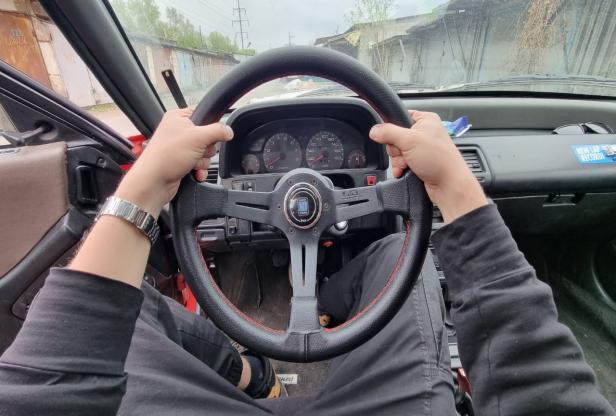

Supplement: Supplemental Information 11 [file peerj-cs-10-2110-s011.zip › keypoint_detection/The 3-Scoped Steering Wheel.v4i.yolov5pytorch/train/images/photo_2022-04-15_10-20-10_jpg.rf.b19fe2c3c61dcd02535a125ea22a412a.jpg]

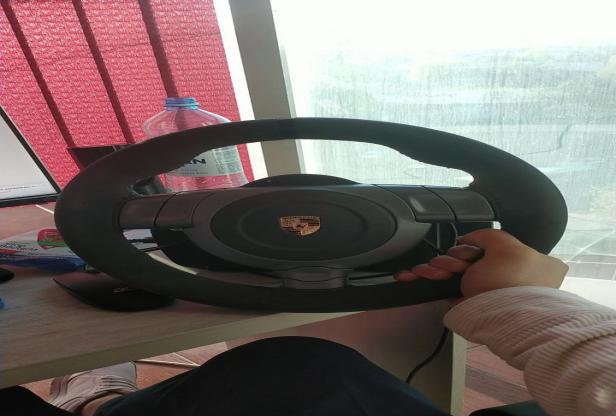

Supplement: Supplemental Information 11 [file peerj-cs-10-2110-s011.zip › keypoint_detection/The 3-Scoped Steering Wheel.v4i.yolov5pytorch/train/images/photo_2022-04-15_18-02-35_jpg.rf.9f36f6fb3661ad5522ad14d338e327a5.jpg]

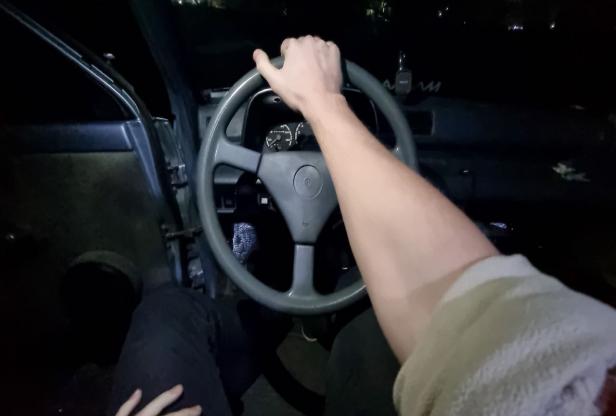

Supplement: Supplemental Information 11 [file peerj-cs-10-2110-s011.zip › keypoint_detection/The 3-Scoped Steering Wheel.v4i.yolov5pytorch/train/images/photo_2022-04-15_21-26-47_jpg.rf.9083ffe13bf5878ab5b897045cfa32bf.jpg]

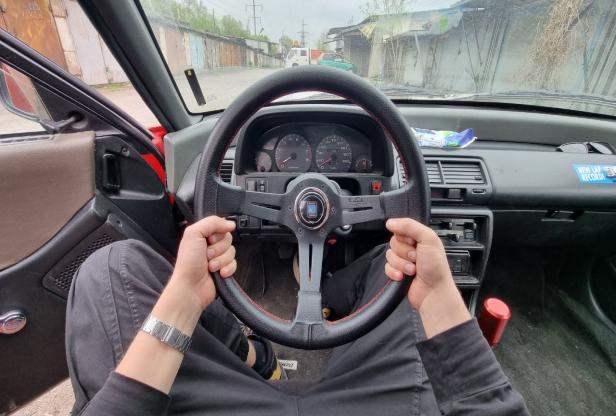

Supplement: Supplemental Information 11 [file peerj-cs-10-2110-s011.zip › keypoint_detection/The 3-Scoped Steering Wheel.v4i.yolov5pytorch/train/images/photo_2022-04-15_10-19-52_jpg.rf.e02c9fd2caf471b03f253fe80e376aad.jpg]

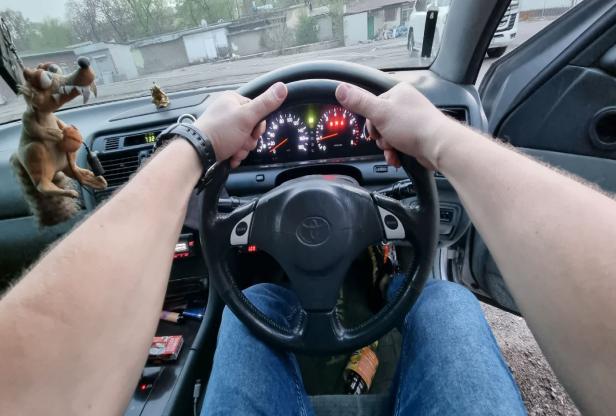

Supplement: Supplemental Information 11 [file peerj-cs-10-2110-s011.zip › keypoint_detection/The 3-Scoped Steering Wheel.v4i.yolov5pytorch/train/images/photo_2022-04-11_21-27-57_jpg.rf.9364915a6476719acc919ce979635162.jpg]

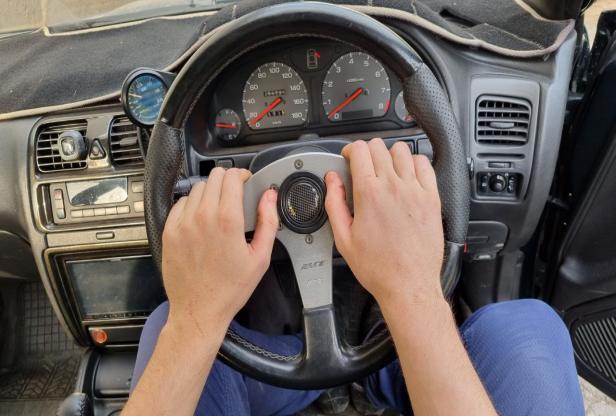

Supplement: Supplemental Information 11 [file peerj-cs-10-2110-s011.zip › keypoint_detection/The 3-Scoped Steering Wheel.v4i.yolov5pytorch/train/images/photo_2022-04-11_21-27-38_jpg.rf.e334515a15019b04e0fc48d285f234b2.jpg]

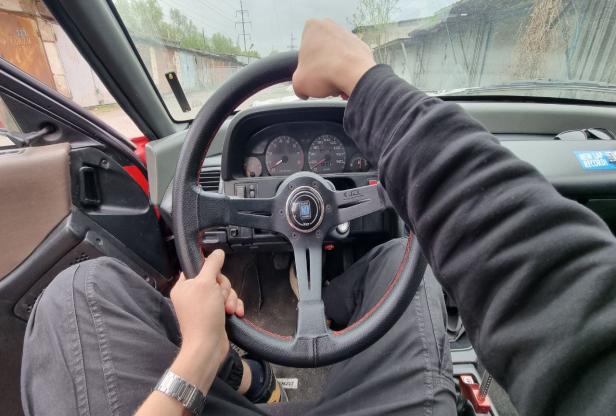

Supplement: Supplemental Information 11 [file peerj-cs-10-2110-s011.zip › keypoint_detection/The 3-Scoped Steering Wheel.v4i.yolov5pytorch/train/images/photo_2022-04-15_10-20-09_jpg.rf.d082b77b74b58ae35bc7d79d9943912a.jpg]

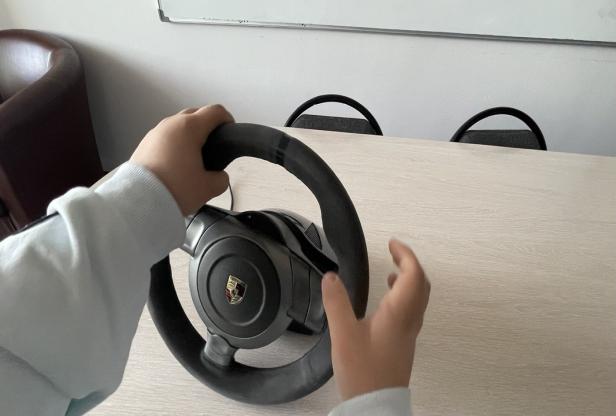

Supplement: Supplemental Information 11 [file peerj-cs-10-2110-s011.zip › keypoint_detection/The 3-Scoped Steering Wheel.v4i.yolov5pytorch/train/images/photo_2023-03-01_11-55-34_jpg.rf.a92fbc610b27a11fa95cb5b8bec36df2.jpg]

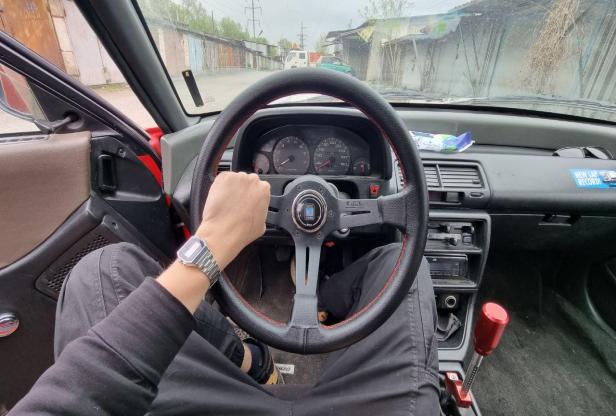

Supplement: Supplemental Information 11 [file peerj-cs-10-2110-s011.zip › keypoint_detection/The 3-Scoped Steering Wheel.v4i.yolov5pytorch/train/images/photo_2022-04-15_10-19-55_jpg.rf.bce1b03e265a589307b65190e57058c2.jpg]

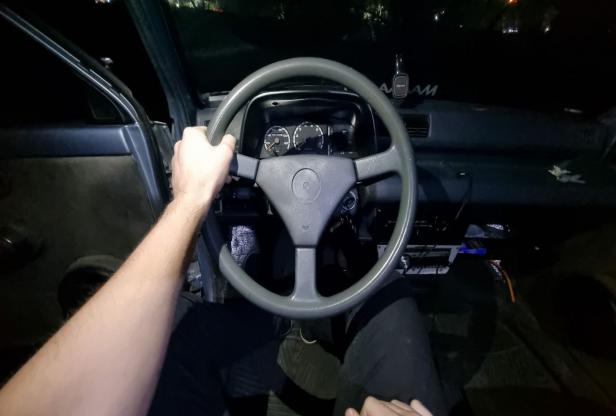

Supplement: Supplemental Information 11 [file peerj-cs-10-2110-s011.zip › keypoint_detection/The 3-Scoped Steering Wheel.v4i.yolov5pytorch/train/images/photo_2022-04-15_21-26-39_jpg.rf.84a838ac1ef60bc2b4070117de0b2c8e.jpg]

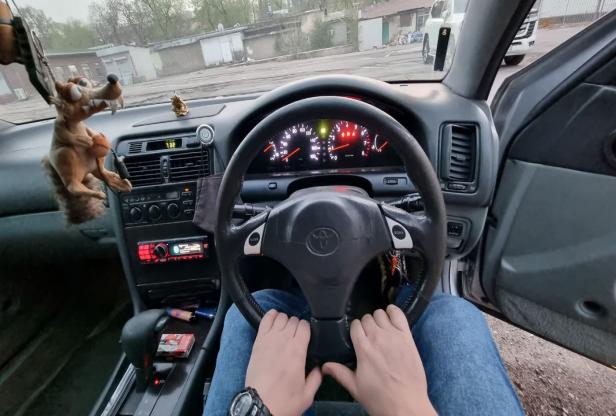

Supplement: Supplemental Information 11 [file peerj-cs-10-2110-s011.zip › keypoint_detection/The 3-Scoped Steering Wheel.v4i.yolov5pytorch/train/images/photo_2022-04-11_21-27-49_jpg.rf.fa28d4aedbf126aea5afaeac9c3c0601.jpg]

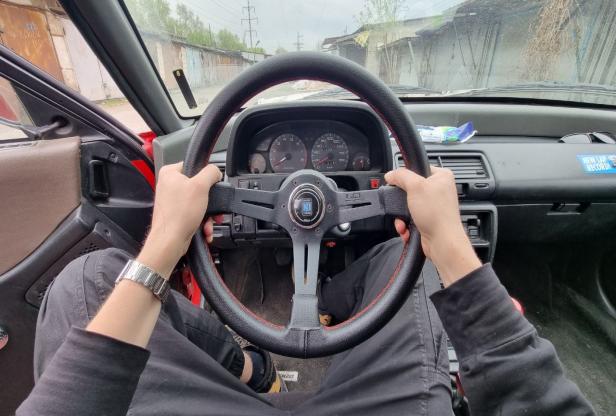

Supplement: Supplemental Information 11 [file peerj-cs-10-2110-s011.zip › keypoint_detection/The 3-Scoped Steering Wheel.v4i.yolov5pytorch/train/images/photo_2022-04-15_10-20-07_jpg.rf.c2225cae76153fae4d8c44c806403060.jpg]

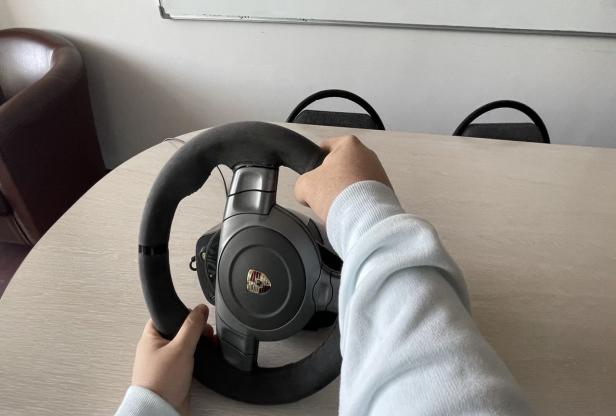

Supplement: Supplemental Information 11 [file peerj-cs-10-2110-s011.zip › keypoint_detection/The 3-Scoped Steering Wheel.v4i.yolov5pytorch/train/images/photo_2023-03-01_11-55-35_jpg.rf.03525c05ae1377e9bf4768935b3b5d55.jpg]

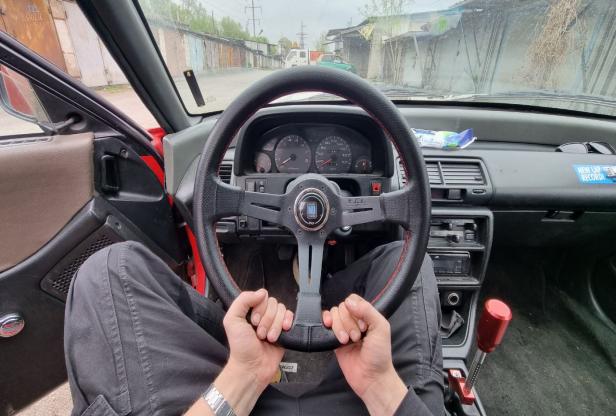

Supplement: Supplemental Information 11 [file peerj-cs-10-2110-s011.zip › keypoint_detection/The 3-Scoped Steering Wheel.v4i.yolov5pytorch/train/images/photo_2022-04-15_10-19-53-2-_jpg.rf.1795deacb70c966ea67d8e45294a72ec.jpg]

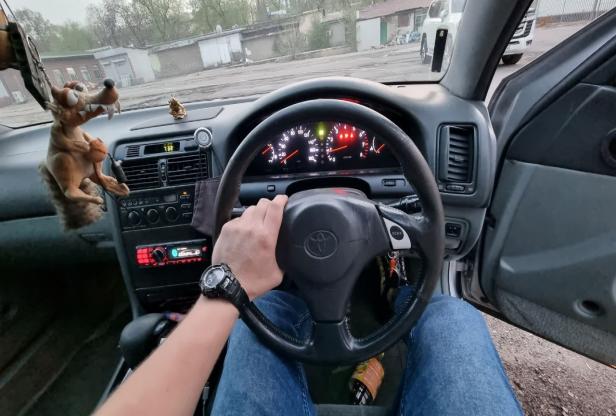

Supplement: Supplemental Information 11 [file peerj-cs-10-2110-s011.zip › keypoint_detection/The 3-Scoped Steering Wheel.v4i.yolov5pytorch/train/images/photo_2022-04-11_21-27-51_jpg.rf.934f593f3f36ea9773e614e09b88cde0.jpg]

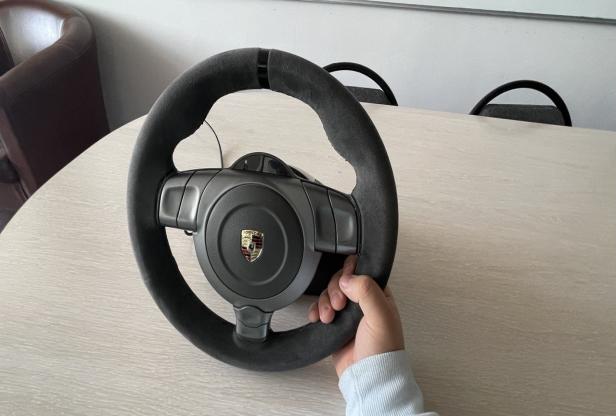

Supplement: Supplemental Information 11 [file peerj-cs-10-2110-s011.zip › keypoint_detection/The 3-Scoped Steering Wheel.v4i.yolov5pytorch/train/images/photo_2023-03-01_11-55-41_jpg.rf.a58e9ff46795e32b9645a8092acc7c89.jpg]

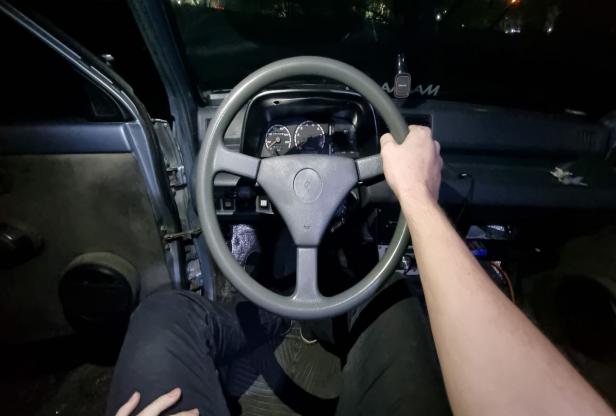

Supplement: Supplemental Information 11 [file peerj-cs-10-2110-s011.zip › keypoint_detection/The 3-Scoped Steering Wheel.v4i.yolov5pytorch/train/images/photo_2022-04-15_21-26-42_jpg.rf.2b74c543f71fa72baaee53d7e169e81f.jpg]

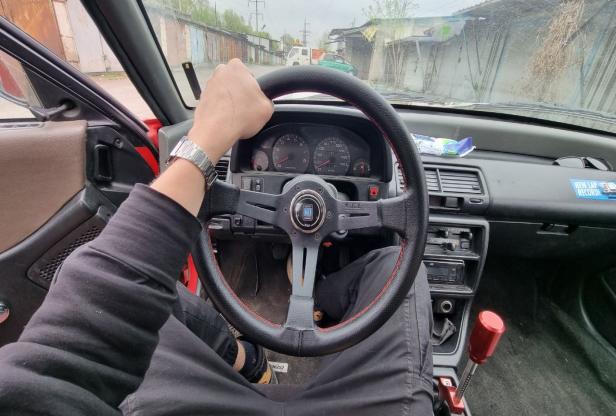

Supplement: Supplemental Information 11 [file peerj-cs-10-2110-s011.zip › keypoint_detection/The 3-Scoped Steering Wheel.v4i.yolov5pytorch/train/images/photo_2022-04-15_10-19-56-2-_jpg.rf.821ea648d68269ebce4351e0b4adbec7.jpg]

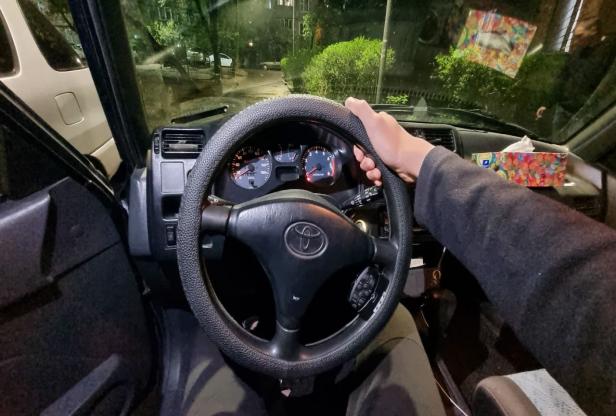

Supplement: Supplemental Information 11 [file peerj-cs-10-2110-s011.zip › keypoint_detection/The 3-Scoped Steering Wheel.v4i.yolov5pytorch/train/images/photo_2022-04-13_10-02-28_jpg.rf.af6eedad24be04d4a8c5769143866953.jpg]

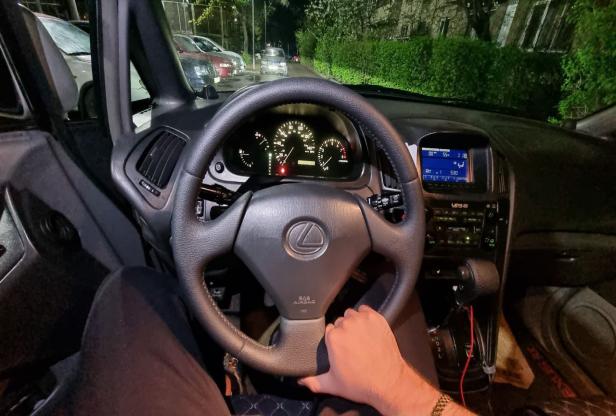

Supplement: Supplemental Information 11 [file peerj-cs-10-2110-s011.zip › keypoint_detection/The 3-Scoped Steering Wheel.v4i.yolov5pytorch/train/images/photo_2022-04-13_10-02-18-2-_jpg.rf.02e7f0334816271af3fad31507731774.jpg]

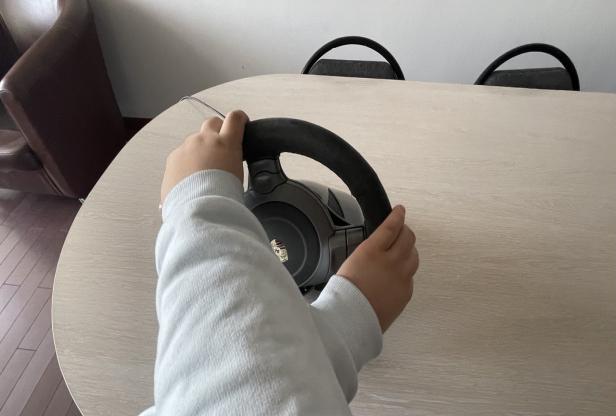

Supplement: Supplemental Information 11 [file peerj-cs-10-2110-s011.zip › keypoint_detection/The 3-Scoped Steering Wheel.v4i.yolov5pytorch/train/images/photo_2023-03-01_11-55-32_jpg.rf.2ddfd6beda2ad52c4abb1ee04e4d01e1.jpg]

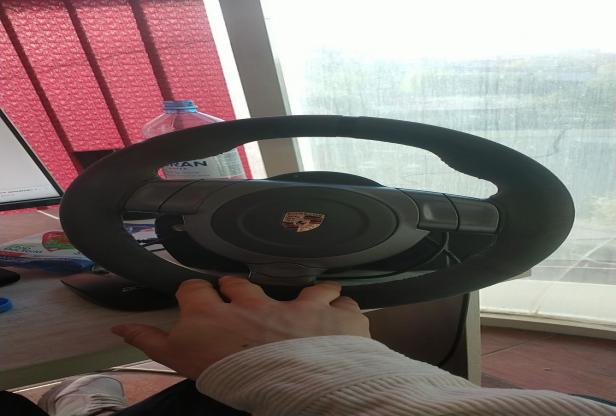

Supplement: Supplemental Information 11 [file peerj-cs-10-2110-s011.zip › keypoint_detection/The 3-Scoped Steering Wheel.v4i.yolov5pytorch/train/images/photo_2022-04-15_18-02-38_jpg.rf.fd04ddb7d75ae4a4a528053172ce0e9d.jpg]

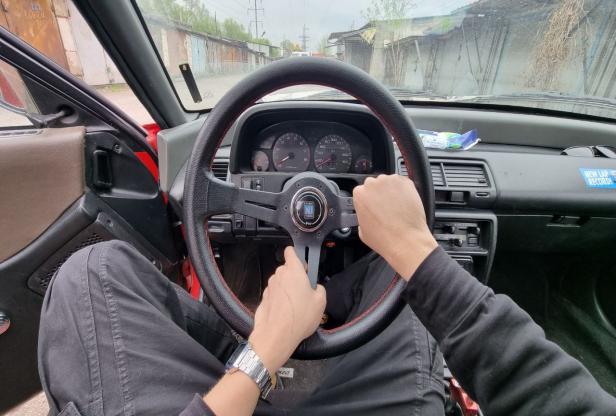

Supplement: Supplemental Information 11 [file peerj-cs-10-2110-s011.zip › keypoint_detection/The 3-Scoped Steering Wheel.v4i.yolov5pytorch/train/images/photo_2022-04-15_10-20-05_jpg.rf.4359b23b88bb506eb927f0dfcfc545c8.jpg]

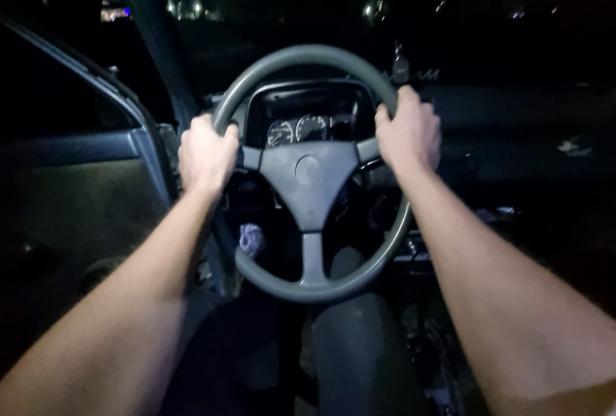

Supplement: Supplemental Information 11 [file peerj-cs-10-2110-s011.zip › keypoint_detection/The 3-Scoped Steering Wheel.v4i.yolov5pytorch/train/images/photo_2022-04-15_21-27-13_jpg.rf.90608cbd9940b05b32a79873005f280b.jpg]

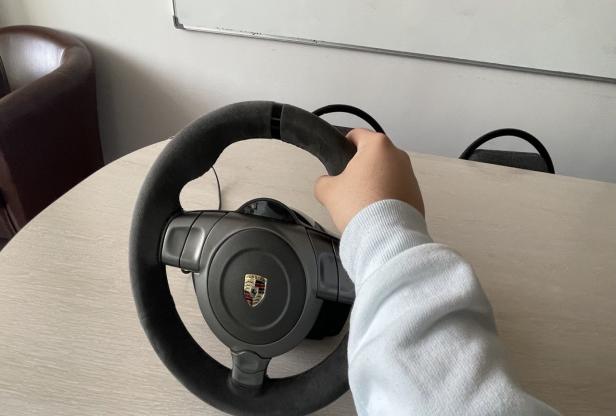

Supplement: Supplemental Information 11 [file peerj-cs-10-2110-s011.zip › keypoint_detection/The 3-Scoped Steering Wheel.v4i.yolov5pytorch/train/images/photo_2023-03-01_11-55-40_jpg.rf.acf48a22a37e9c232202c97451d0411a.jpg]

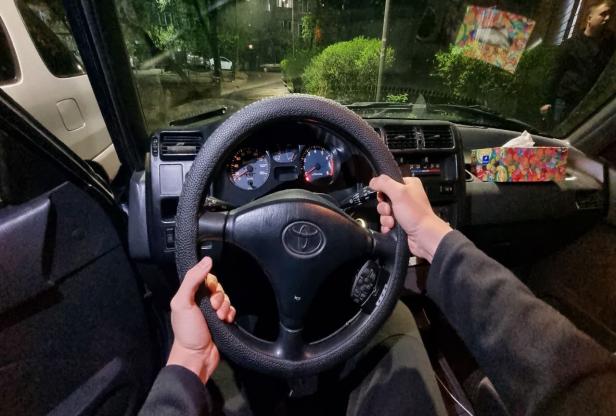

Supplement: Supplemental Information 11 [file peerj-cs-10-2110-s011.zip › keypoint_detection/The 3-Scoped Steering Wheel.v4i.yolov5pytorch/train/images/photo_2022-04-13_10-02-30_jpg.rf.e50dafd8e88718bf6d2048cda7fcfa61.jpg]

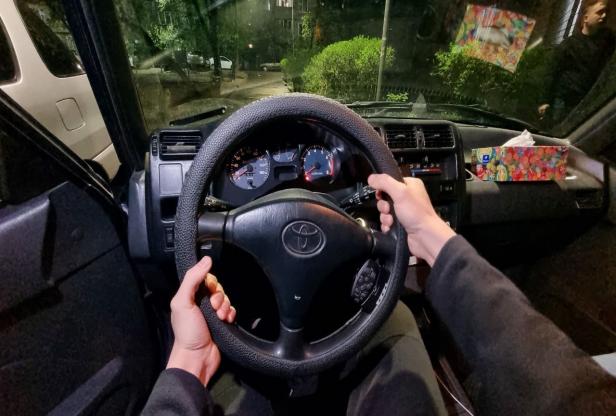

Supplement: Supplemental Information 11 [file peerj-cs-10-2110-s011.zip › keypoint_detection/The 3-Scoped Steering Wheel.v4i.yolov5pytorch/train/images/photo_2022-04-13_10-02-31_jpg.rf.2e14a6283f601c5362175ffbb29224de.jpg]

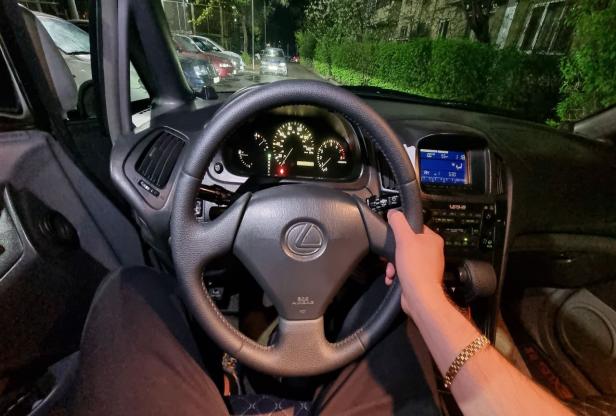

Supplement: Supplemental Information 11 [file peerj-cs-10-2110-s011.zip › keypoint_detection/The 3-Scoped Steering Wheel.v4i.yolov5pytorch/train/images/photo_2022-04-13_10-02-19_jpg.rf.8090f47012ca820596da94046b1ff0b4.jpg]

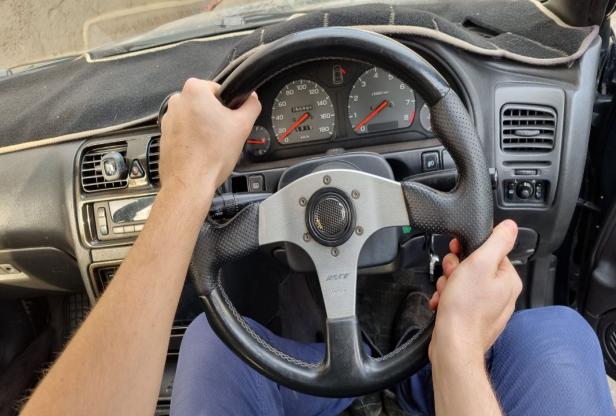

Supplement: Supplemental Information 11 [file peerj-cs-10-2110-s011.zip › keypoint_detection/The 3-Scoped Steering Wheel.v4i.yolov5pytorch/train/images/photo_2022-04-11_21-27-40_jpg.rf.1bae0e00f6a0f80fbea1ddb0063a2fb1.jpg]

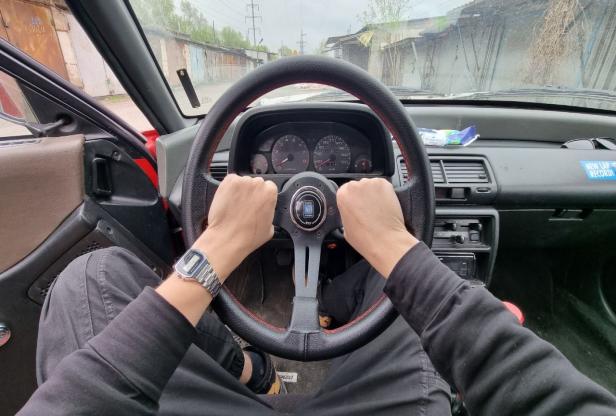

Supplement: Supplemental Information 11 [file peerj-cs-10-2110-s011.zip › keypoint_detection/The 3-Scoped Steering Wheel.v4i.yolov5pytorch/train/images/photo_2022-04-15_10-20-06_jpg.rf.fad23583a8f73d59f62cfc5969a8a103.jpg]

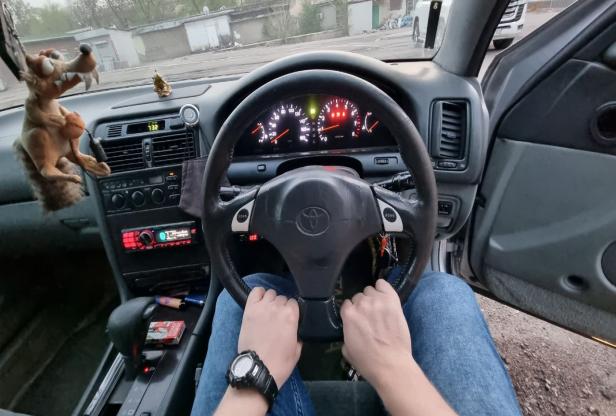

Supplement: Supplemental Information 11 [file peerj-cs-10-2110-s011.zip › keypoint_detection/The 3-Scoped Steering Wheel.v4i.yolov5pytorch/train/images/photo_2022-04-11_21-27-56_jpg.rf.6cd3612ffe3957fc04a3bc64cbab48c6.jpg]

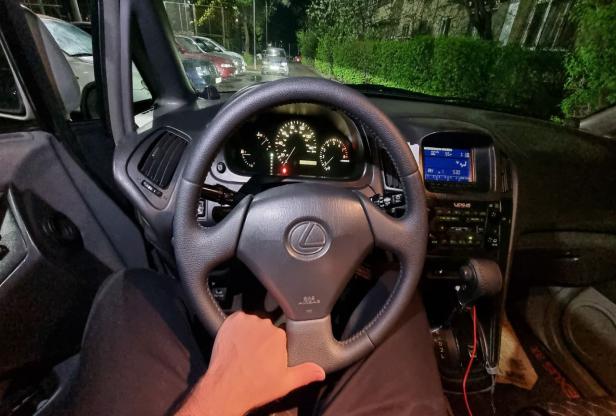

Supplement: Supplemental Information 11 [file peerj-cs-10-2110-s011.zip › keypoint_detection/The 3-Scoped Steering Wheel.v4i.yolov5pytorch/train/images/photo_2022-04-13_10-02-18_jpg.rf.21d49f70f44509e5b8b53502ebc46b41.jpg]

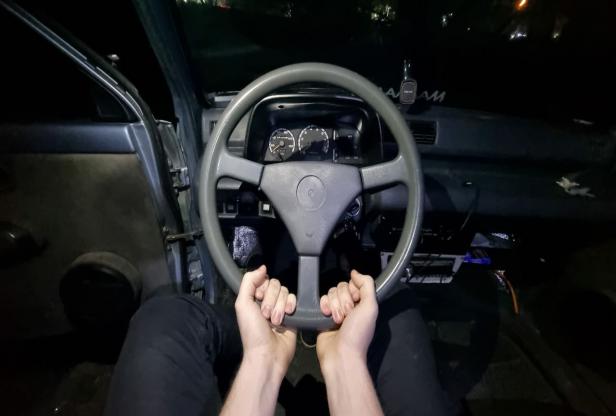

Supplement: Supplemental Information 11 [file peerj-cs-10-2110-s011.zip › keypoint_detection/The 3-Scoped Steering Wheel.v4i.yolov5pytorch/train/images/photo_2022-04-15_21-26-29_jpg.rf.e97c5afb2dbc5b0a0ced71aa66ce25f3.jpg]

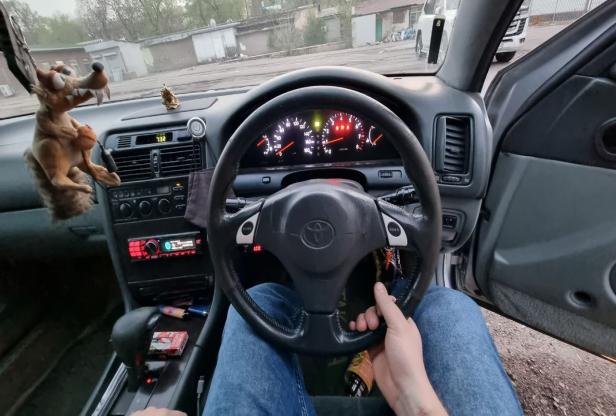

Supplement: Supplemental Information 11 [file peerj-cs-10-2110-s011.zip › keypoint_detection/The 3-Scoped Steering Wheel.v4i.yolov5pytorch/train/images/photo_2022-04-11_21-27-54_jpg.rf.a44c5473a17c70b311185df5a96d36aa.jpg]

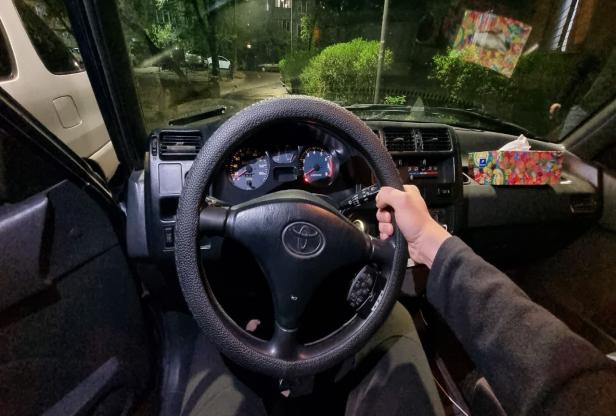

Supplement: Supplemental Information 11 [file peerj-cs-10-2110-s011.zip › keypoint_detection/The 3-Scoped Steering Wheel.v4i.yolov5pytorch/train/images/photo_2022-04-13_10-02-29_jpg.rf.85ba31a843d49442942f6315d67c6552.jpg]

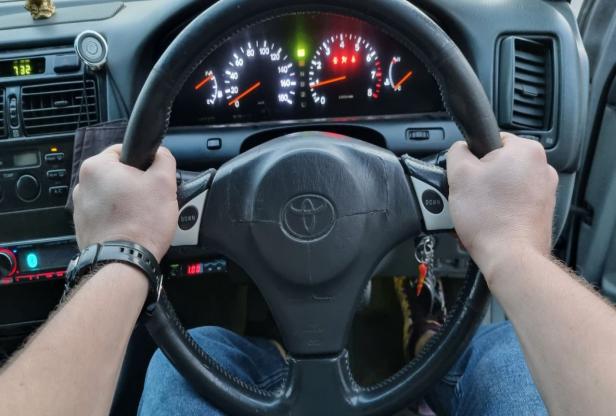

Supplement: Supplemental Information 11 [file peerj-cs-10-2110-s011.zip › keypoint_detection/The 3-Scoped Steering Wheel.v4i.yolov5pytorch/train/images/photo_2022-04-11_21-28-00_jpg.rf.5aa458b7e10c33c1f1039173ad6269b7.jpg]

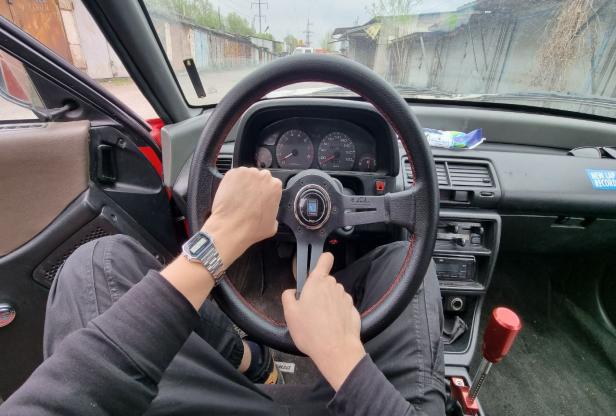

Supplement: Supplemental Information 11 [file peerj-cs-10-2110-s011.zip › keypoint_detection/The 3-Scoped Steering Wheel.v4i.yolov5pytorch/train/images/photo_2022-04-15_10-20-04_jpg.rf.dc3d0cbad03f4b8867c8a3929e0cd27a.jpg]

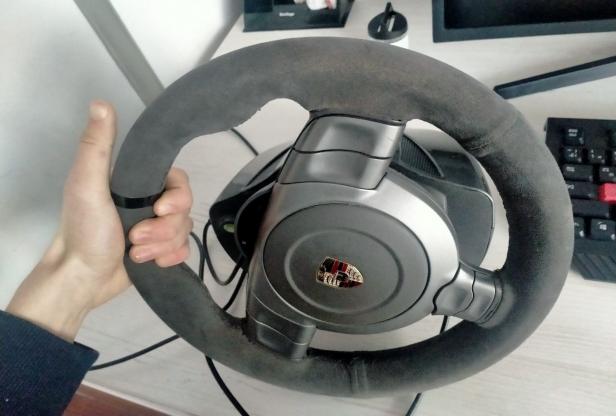

Supplement: Supplemental Information 11 [file peerj-cs-10-2110-s011.zip › keypoint_detection/The 3-Scoped Steering Wheel.v4i.yolov5pytorch/train/images/photo_2022-03-14_16-00-39_jpg.rf.8ba7b292ab8b9252af666753f28bb200.jpg]

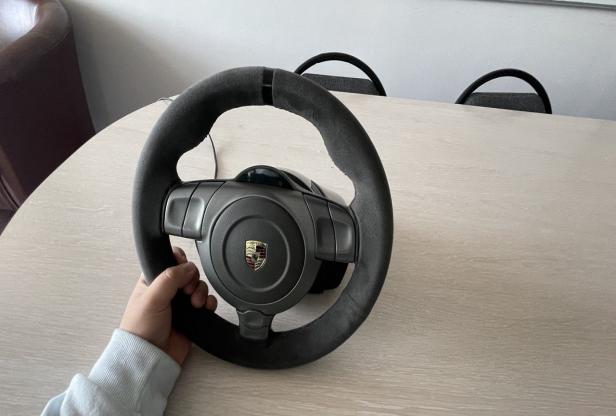

Supplement: Supplemental Information 11 [file peerj-cs-10-2110-s011.zip › keypoint_detection/The 3-Scoped Steering Wheel.v4i.yolov5pytorch/train/images/photo_2023-03-01_11-55-38_jpg.rf.6888afd17efff95af999c4e1a26f633e.jpg]

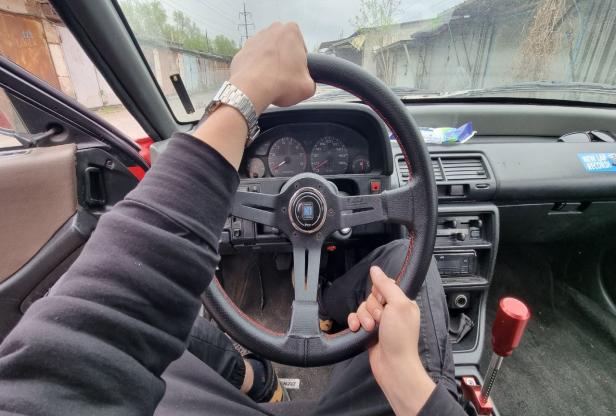

Supplement: Supplemental Information 11 [file peerj-cs-10-2110-s011.zip › keypoint_detection/The 3-Scoped Steering Wheel.v4i.yolov5pytorch/train/images/photo_2022-04-15_10-20-08_jpg.rf.2584774c8986bad6f7f545c08da65758.jpg]

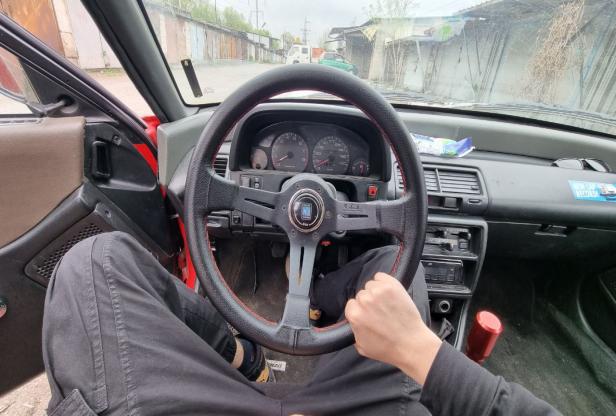

Supplement: Supplemental Information 11 [file peerj-cs-10-2110-s011.zip › keypoint_detection/The 3-Scoped Steering Wheel.v4i.yolov5pytorch/train/images/photo_2022-04-15_10-19-58_jpg.rf.f3f2415249877c105d90db8efbc46170.jpg]

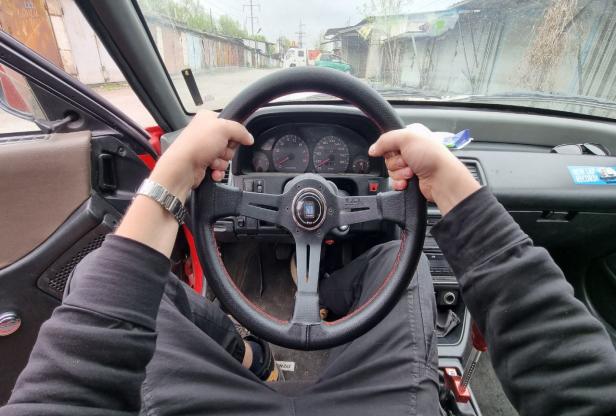

Supplement: Supplemental Information 11 [file peerj-cs-10-2110-s011.zip › keypoint_detection/The 3-Scoped Steering Wheel.v4i.yolov5pytorch/train/images/photo_2022-04-15_10-19-53_jpg.rf.0de2848ebb665169f940c71055de03a8.jpg]

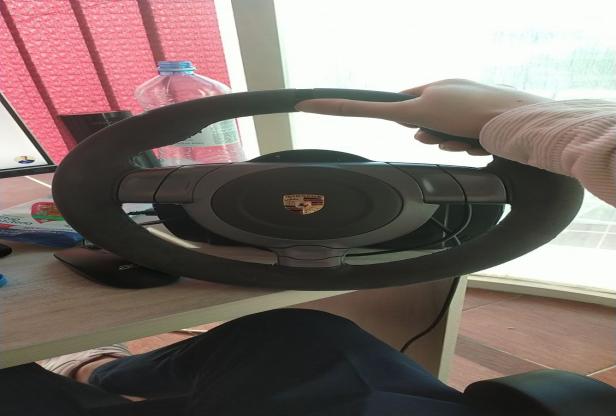

Supplement: Supplemental Information 11 [file peerj-cs-10-2110-s011.zip › keypoint_detection/The 3-Scoped Steering Wheel.v4i.yolov5pytorch/train/images/photo_2022-04-15_18-02-32_jpg.rf.ee9a7ec645d16ceaeb467703eb160f41.jpg]

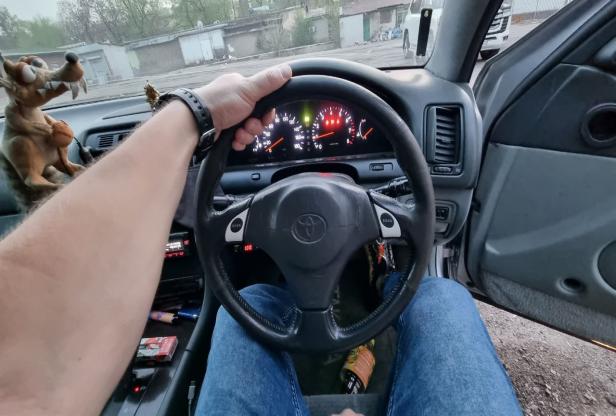

Supplement: Supplemental Information 11 [file peerj-cs-10-2110-s011.zip › keypoint_detection/The 3-Scoped Steering Wheel.v4i.yolov5pytorch/train/images/photo_2022-04-11_21-27-58_jpg.rf.16bf14bc806c39dbdbd1987c97e102d5.jpg]

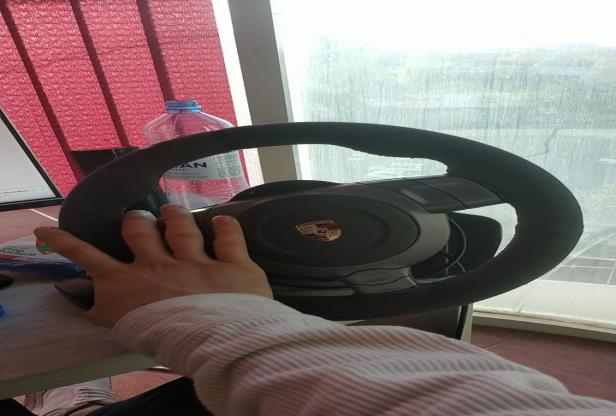

Supplement: Supplemental Information 11 [file peerj-cs-10-2110-s011.zip › keypoint_detection/The 3-Scoped Steering Wheel.v4i.yolov5pytorch/train/images/photo_2022-04-15_18-02-37_jpg.rf.a27b155a99c31d79d893bd632e62c6ea.jpg]

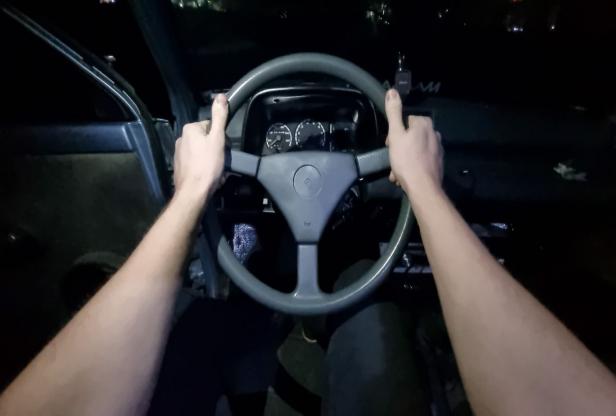

Supplement: Supplemental Information 11 [file peerj-cs-10-2110-s011.zip › keypoint_detection/The 3-Scoped Steering Wheel.v4i.yolov5pytorch/train/images/photo_2022-04-15_21-26-54_jpg.rf.6072987ecee21e60f3eb127f31693570.jpg]

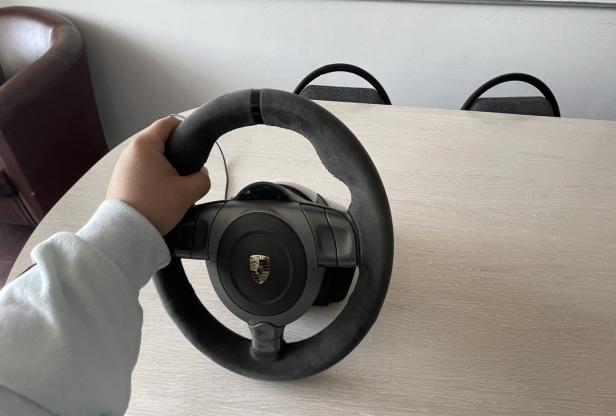

Supplement: Supplemental Information 11 [file peerj-cs-10-2110-s011.zip › keypoint_detection/The 3-Scoped Steering Wheel.v4i.yolov5pytorch/train/images/photo_2023-03-01_11-55-37_jpg.rf.c20e5ab65ef0817fd8f81c4e7313a286.jpg]

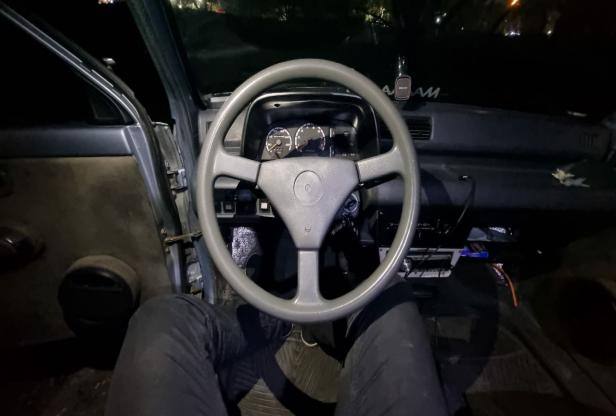

Supplement: Supplemental Information 11 [file peerj-cs-10-2110-s011.zip › keypoint_detection/The 3-Scoped Steering Wheel.v4i.yolov5pytorch/train/images/photo_2022-04-15_21-26-36_jpg.rf.c6d722f638094c0a7993438615e21ba6.jpg]

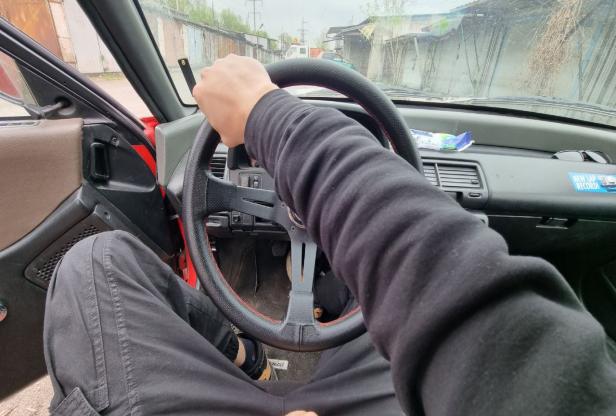

Supplement: Supplemental Information 11 [file peerj-cs-10-2110-s011.zip › keypoint_detection/The 3-Scoped Steering Wheel.v4i.yolov5pytorch/train/images/photo_2022-04-15_10-19-59_jpg.rf.5a6a5f724d531e0152fe4d48dd9e42f5.jpg]

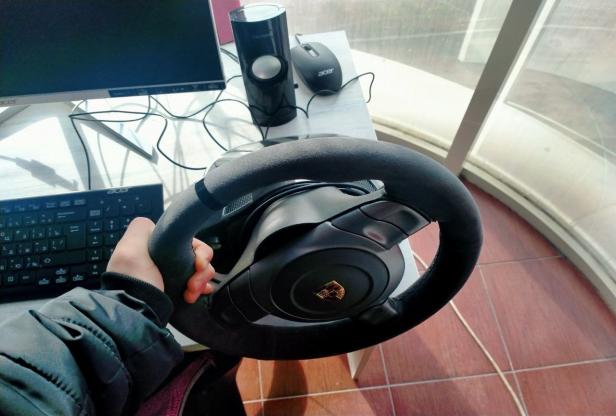

Supplement: Supplemental Information 11 [file peerj-cs-10-2110-s011.zip › keypoint_detection/The 3-Scoped Steering Wheel.v4i.yolov5pytorch/train/images/photo_2022-03-14_16-00-42_jpg.rf.b0773b4ef2ae03013aaec6e9ca9cbe94.jpg]

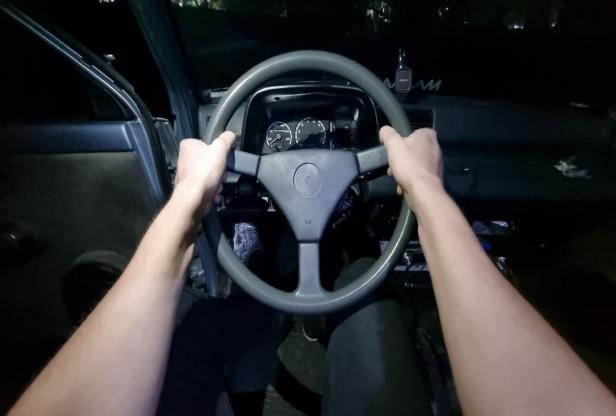

Supplement: Supplemental Information 11 [file peerj-cs-10-2110-s011.zip › keypoint_detection/The 3-Scoped Steering Wheel.v4i.yolov5pytorch/train/images/photo_2022-04-15_21-26-56_jpg.rf.4444d40e0cce0047d5cfc23cd4732e15.jpg]

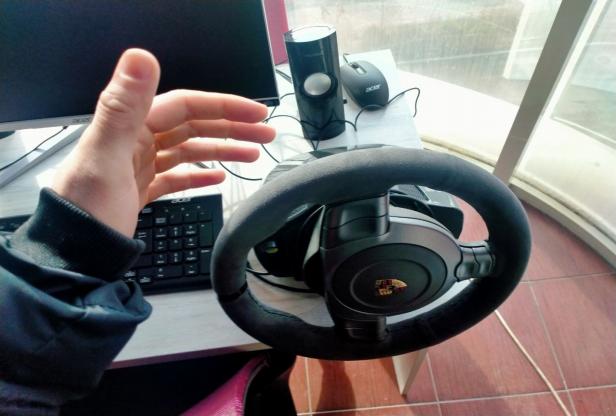

Supplement: Supplemental Information 11 [file peerj-cs-10-2110-s011.zip › keypoint_detection/The 3-Scoped Steering Wheel.v4i.yolov5pytorch/train/images/photo_2022-03-14_16-00-32_jpg.rf.a000d7a0f322740447603c381a1a70c7.jpg]

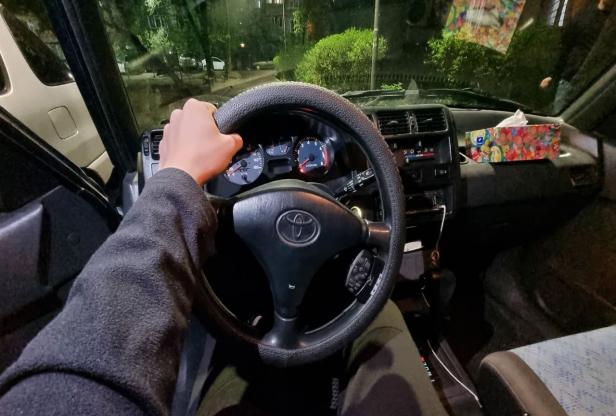

Supplement: Supplemental Information 11 [file peerj-cs-10-2110-s011.zip › keypoint_detection/The 3-Scoped Steering Wheel.v4i.yolov5pytorch/train/images/photo_2022-04-13_10-02-32_jpg.rf.f2fb7dba00bf286b950668d4e8b2dff0.jpg]

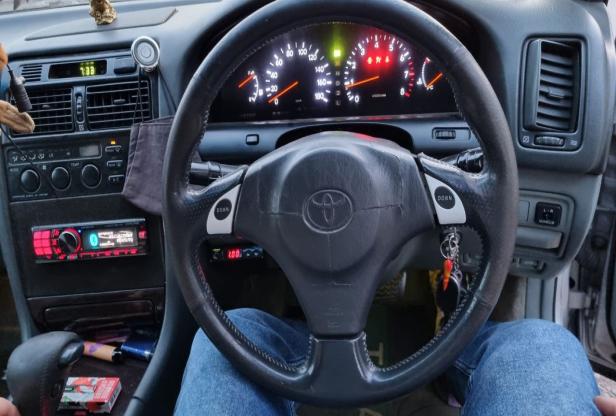

Supplement: Supplemental Information 11 [file peerj-cs-10-2110-s011.zip › keypoint_detection/The 3-Scoped Steering Wheel.v4i.yolov5pytorch/train/images/photo_2022-04-11_21-27-46_jpg.rf.f0e53aca6d9c2ccc0ed15f6013bdece6.jpg]

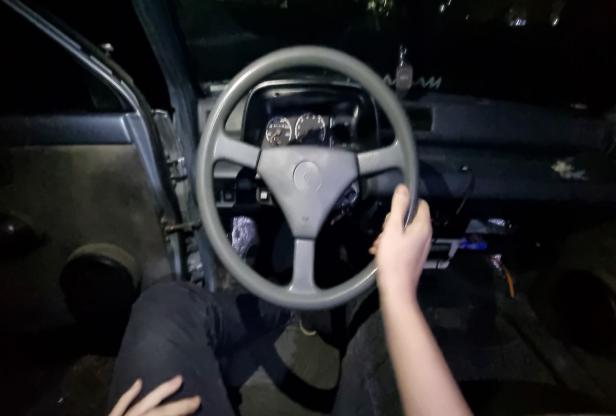

Supplement: Supplemental Information 11 [file peerj-cs-10-2110-s011.zip › keypoint_detection/The 3-Scoped Steering Wheel.v4i.yolov5pytorch/train/images/photo_2022-04-15_21-26-45_jpg.rf.db6e993eb4edc57c0d33c606f3f0c251.jpg]

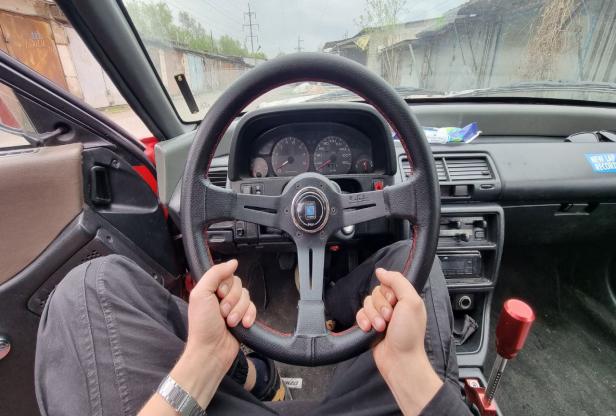

Supplement: Supplemental Information 11 [file peerj-cs-10-2110-s011.zip › keypoint_detection/The 3-Scoped Steering Wheel.v4i.yolov5pytorch/train/images/photo_2022-04-15_10-20-07-2-_jpg.rf.0af35692ba6d3301be5d25eb9c2a7881.jpg]

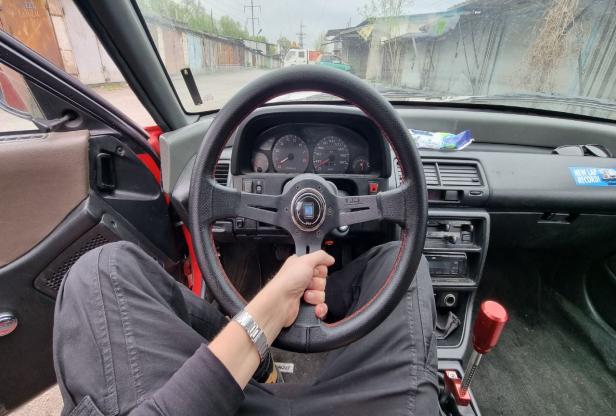

Supplement: Supplemental Information 11 [file peerj-cs-10-2110-s011.zip › keypoint_detection/The 3-Scoped Steering Wheel.v4i.yolov5pytorch/train/images/photo_2022-04-15_10-19-54_jpg.rf.3a76e9270c110f5f83244dd2ef3540fb.jpg]

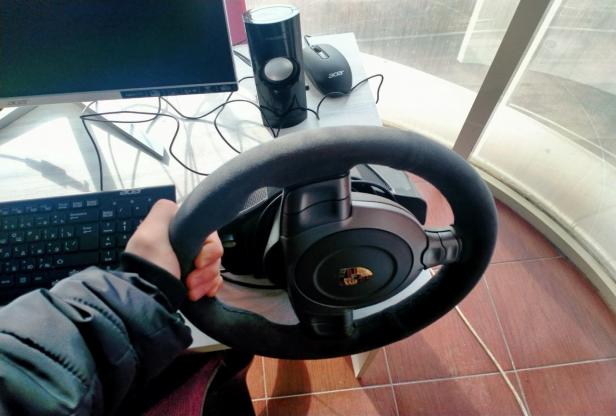

Supplement: Supplemental Information 11 [file peerj-cs-10-2110-s011.zip › keypoint_detection/The 3-Scoped Steering Wheel.v4i.yolov5pytorch/train/images/photo_2022-03-14_16-00-44_jpg.rf.d9d64ecd8d3913f6c8e3b7129d400c1d.jpg]
